# Regulation of Postabsorptive and Postprandial Glucose Metabolism by Insulin-Dependent and Insulin-Independent Mechanisms: An Integrative Approach

**DOI:** 10.3390/nu13010159

**Published:** 2021-01-06

**Authors:** George D. Dimitriadis, Eirini Maratou, Aikaterini Kountouri, Mary Board, Vaia Lambadiari

**Affiliations:** 1 Sector of Medicine, Medical School, National and Kapodistrian University of Athens, 15772 Athens, Greece; 2 Department of Clinical Biochemistry, Medical School, National and Kapodistrian University of Athens, 15772 Athens, Greece; maratou@hotmail.com; 3 Department of Clinical Biochemistry, Medical School, “Attikon” University Hospital, Rimini 1, 12462 Chaidari, Greece; 4 Research Institute and Diabetes Center, 2nd Department of Internal Medicine, “Attikon” University Hospital, 1 Rimini Street, 12542 Haidari, Greece; katerinak90@hotmail.com (A.K.); vlambad@otenet.gr (V.L.); 5 St. Hilda’s College, University of Oxford, Cowley, Oxford OX4 1DY, UK; mary.board@st-hildas.ox.ac.uk

**Keywords:** postabsorptive postprandial glucose metabolism, fasting, insulin action secretion, liver, muscle, adipose tissue, incretins, meal sequence

## Abstract

Glucose levels in blood must be constantly maintained within a tight physiological range to sustain anabolism. Insulin regulates glucose homeostasis via its effects on glucose production from the liver and kidneys and glucose disposal in peripheral tissues (mainly skeletal muscle). Blood levels of glucose are regulated simultaneously by insulin-mediated rates of glucose production from the liver (and kidneys) and removal from muscle; adipose tissue is a key partner in this scenario, providing nonesterified fatty acids (NEFA) as an alternative fuel for skeletal muscle and liver when blood glucose levels are depleted. During sleep at night, the gradual development of insulin resistance, due to growth hormone and cortisol surges, ensures that blood glucose levels will be maintained within normal levels by: (a) switching from glucose to NEFA oxidation in muscle; (b) modulating glucose production from the liver/kidneys. After meals, several mechanisms (sequence/composition of meals, gastric emptying/intestinal glucose absorption, gastrointestinal hormones, hyperglycemia mass action effects, insulin/glucagon secretion/action, de novo lipogenesis and glucose disposal) operate in concert for optimal regulation of postprandial glucose fluctuations. The contribution of the liver in postprandial glucose homeostasis is critical. The liver is preferentially used to dispose over 50% of the ingested glucose and restrict the acute increases of glucose and insulin in the bloodstream after meals, thus protecting the circulation and tissues from the adverse effects of marked hyperglycemia and hyperinsulinemia.

## 1. Introduction

Food has major effects on physical health. Although there is an obvious need for energy to perform physical activities, energy requirements persist during rest. The substrates used by tissues as energy sources are carbohydrates, fat, and proteins. Although the energy yield of 1 g of fat is more than twice that of 1 g of carbohydrate or protein, tissues depend on glucose as the major energy source. With regard to the brain and red blood cells, glucose is the only fuel they use; therefore, they rely on the bloodstream to provide a constant supply [1].

Insulin plays a primary role in the regulation of glucose homeostasis via its effects on insulin-sensitive tissues: blood levels of glucose are regulated simultaneously by the rates of glucose production from the liver (and kidneys), and the rates of its removal from peripheral tissues (mainly skeletal muscle). Adipose tissue is a key partner in this scenario, providing nonesterified fatty acids (NEFA) as an alternative fuel for skeletal muscle, liver, and kidneys when blood glucose levels are depleted. These mechanisms are supported by balanced adjustments in insulin secretion to keep the metabolic system under tight control. The changes in insulin secretion and action are highly coordinated by the central nervous system (CNS) to ensure appropriate substrate switching between tissues to meet metabolic needs, such as in the postabsorptive to postprandial transition [2,3,4,5].

## 2. Physiology of Insulin Effects on Target Tissues 

### 2.1. Liver

The liver plays a key role in glucose homeostasis. Its main metabolic function is to store glucose as glycogen after a meal and release it into the bloodstream (via glycogenolysis and gluconeogenesis) when required to maintain a constant concentration of glucose under any circumstances [6]. In hepatocytes, glucose transport does not depend on insulin. The high Km of GLUT2 glucose transporters (~20 mM) and glucokinase (~12 mM) allow glucose to enter the cells at a rate proportional to the concentration of extracellular glucose, facilitating glucose clearance following a meal [7,8]. Hepatic glucose production is inhibited by hyperinsulinemia and hyperglycemia [9,10].

Liver glycogen provides an immediately available reserve of glucose to maintain blood glucose concentrations, such as during hypoglycemia, fasting, or exercise [6,11,12]. Gluconeogenesis is a complex, branched pathway and occurs only in the liver and kidney cortex [13]. Control can be achieved by variations in the blood concentrations of the end product (glucose) and of the precursors by insulin and anti-insulin hormones and by central mechanisms involving the action of insulin at the hypothalamus and other parts of the brain [14,15,16,17,18]. The major precursors for liver gluconeogenesis are lactate, pyruvate, glycerol, and amino acids of which alanine and glutamine are quantitatively important [19].

A critical factor to consider in the regulation of the rate of gluconeogenesis is the supply of NEFA to the liver [20,21]. Increased rates of NEFA oxidation in hepatocytes activate key gluconeogenic enzymes and stimulate gluconeogenesis. Inhibition of lipolysis in the adipose tissue by insulin will therefore decrease the flow of both a stimulator (NEFA) and a substrate (glycerol) of gluconeogenesis to the liver, thus decreasing the rate of hepatic glucose production [20,21,22,23,24,25].

Glucagon and adrenaline mainly, but on a chronic basis also cortisol and growth hormone, compete with insulin and increase hepatic glucose production through an increase in glycogenolysis and gluconeogenesis [26,27,28,29]. Adrenaline, cortisol, and growth hormone also increase gluconeogenesis by an indirect mechanism, via an increase in lipolysis in adipose tissue and supply of glycerol and NEFA to the liver [30]. These effects of growth hormone and cortisol are of major importance in developing a transient insulin resistant state in the early morning hours during sleep, which helps to maintain euglycemia (see below).

### 2.2. Kidneys

The kidneys also express all the enzymes of the gluconeogenic pathway with the main gluconeogenic substrates being lactate, glutamine, and glycerol, whereas alanine is preferentially used by the liver rather than the kidneys [31]. Physiologic concentrations of insulin suppress renal gluconeogenesis and glucose production to approximately the same extent as in the liver, probably by intrarenal effects rather than by reducing substrate delivery [32,33,34]. Because NEFA have been shown to stimulate renal gluconeogenesis, the suppression of renal glucose production by insulin may be mediated, at least in part, by the decrease in adipose tissue lipolysis and plasma levels of NEFA when insulin concentrations are increased [33].

### 2.3. Skeletal Muscle and Adipose Tissue

Glucose transport is an important step in cell metabolism because it controls the rate of glucose utilization. In skeletal muscle and adipose tissue, the glucose transporter isoforms expressed are GLUT1, GLUT3, and GLUT4 [35]. In the postprandial state, insulin increases the rate of glucose transport mainly by stimulating the translocation of GLUT4 isoforms (Km ~5 mM) from the intracellular pool to the cell membrane [35,36]. In the fasting (basal) state, glucose transport is independent of insulin and is facilitated by the GLUT1 glucose transporters the low Km of which (~2 mM) is well suited for their function, to ensure basal glucose uptake [35].

Insulin increases the rate of glycolysis by increasing glucose transport and by regulating the activities of hexokinase and 6-phosphofructokinase [37,38,39,40]. Stimulation of glucose transport and the activities of these two enzymes by insulin, which leads to an enhanced rate of glycolysis, is of fundamental metabolic importance [2] for the following reasons: (a) when glycogen store in muscle is replete, the glucose taken up is converted to lactate, to maintain enhanced glucose utilization; (b) lactate produced and released by muscle (and adipose tissue) is taken up by the liver and converted to glycogen (known as the “indirect pathway” of glycogen synthesis) [41,42,43].

Lactate is produced by several tissues, but of these, only muscle and adipose tissue are sensitive to insulin and, therefore, subject to regulation [44,45]. The conversion of glucose to lactate in muscle and adipose tissue and the conversion to glucose in the liver represent a cyclic flow of carbon (Cori cycle) [46]. This can be described as an interorgan substrate cycle by analogy with intracellular substrate cycles [47]. It may have greater physiologic significance than just that of a carbon link between peripheral tissues (such as muscle and adipose tissue) and the liver. By maintaining a continuous flux, the Cori cycle provides a dynamic “buffer” of lactate in which its concentration remains relatively constant both in the tissues and the bloodstream [2]. The benefit of this is that it can be used by tissues whenever required for oxidation or for anabolic purposes [44,46].

Although skeletal muscle can store glucose as glycogen, in contrast to the liver, this tissue cannot release free glucose since it lacks the enzyme glucose 6-phosphatase. However, muscle can supply carbon for glucose production via the glucose-alanine cycle [48]. Alanine is a product of glycolysis in this tissue and is formed by adding an amino group from the metabolism of other amino acids, to pyruvate [49]. The transportation of alanine to the liver and its conversion to glucose via gluconeogenesis provides an effective mechanism to maintain blood glucose levels during fasting by using the large protein reserve in skeletal muscle [50].

Adipose tissue is more than just a passive repository for excess energy. Adipocytes secrete many hormones and cytokines which can affect energy homeostasis and the sensitivity of tissues to insulin [51]. Moreover, the processes of fat storage and mobilization are themselves regulated in a highly coordinated manner, with minute-to-minute control and rapid shifts in metabolic flux such as, for example, in the postabsorptive to postprandial transition [52,53]. The role of adipose tissue in buffering the level and flux of NEFA in the circulation in the fasting and postprandial period is crucial and has been considered analogous to the buffering of the level and flux of glucose by liver and muscle [54]. Adipose tissue provides its buffering action by regulation of the release of NEFA into the circulation according to the conditions of feeding or fasting through a change in the activity of the enzyme hormone-sensitive lipase (HSL) [55]. In addition, it also increases the rate of triglyceride clearance through an increase in the activity of the enzyme lipoprotein lipase (LPL) [56]. The activities of HSL and LPL are decreased and increased, respectively, by insulin [55,56].

The major store of triglycerides in the body is present in adipose tissue and it is mobilized in the form of NEFA and glycerol, which are then carried to other tissues via the bloodstream. Muscle can oxidize NEFA derived from adipose tissue and obtain energy. However, fatty acid oxidation in this tissue does more than provide energy. It provides a regulatory mechanism by which insulin and anti-insulin hormones can modify the rate and fate of glucose metabolism in muscle [2]. Thus, NEFA oxidation decreases the rates of glucose utilization and oxidation. In addition, glucose decreases the rate of NEFA oxidation, so that there is a reciprocal relationship between the oxidation of these two fuels; this control mechanism is known as the “glucose/fatty acid cycle” [57]. One principal point in this mechanism is that the pathway for fatty acid oxidation in muscle (β-oxidation and the Krebs cycle) depends upon the rate of lipolysis in adipose tissue and the ability of this tissue to control the blood concentrations of NEFA [58]. Evidence from human experiments has shown that it is not the increase in fat oxidation that inhibits indirectly insulin-stimulated glucose uptake, but instead, this abnormality results from accumulation of fatty acid metabolites in muscle cells, such as diacylglycerol and ceramides, which interfere with insulin signaling, thus leading to a failure of insulin to stimulate glucose transport [59,60].

Insulin inhibits adipose tissue HSL activity, thus decreasing the rate of mobilization of NEFA from adipose tissue, reducing blood levels of NEFA and leading to a greater rate of glucose utilization by muscle [20,21]. Given that muscle represents a large proportion of total body mass [61], insulin causes a substantial switch from fat to carbohydrate oxidation for the body’s tissues [62]. This is a more effective regulatory mechanism than that of glucose alone and illustrates the role in integrating fat and carbohydrate metabolism across tissues achieved by insulin signaling, permitting metabolism to adapt to changes in energy requirements under all circumstances [63].

Insulin affects vascular endothelium and increases muscle and adipose tissue blood flow by increasing vasodilatation and capillary recruitment [64,65,66]. Insulin-mediated increases in blood flow and insulin’s effects on tissue glucose uptake and metabolism are tightly coupled processes and, therefore, important determinants of tissue sensitivity to insulin [64,67]. In skeletal muscle, the increase in blood flow after a meal or during exercise increases the delivery of substrates for metabolism [63,68,69]. In the adipose tissue, the postprandial increases in the rates of blood flow by insulin are critical for the clearance of NEFA from the bloodstream, and hence facilitate insulin-stimulated glucose utilization in skeletal muscle [63,67,70].

## 3. The Postabsorptive (Fasting) State

This term refers to the period of 6–12 h following a meal until the next one. In a normal weight individual, blood glucose concentrations are maintained usually at ~80–90 mg/dL and insulin levels at ~7–10 μU/mL [71]. Under these steady-state conditions insulin secretion is kept at its basal rates of secretion, there is no net storage of glucose and the rates of endogenous glucose production (~2 mg/kg/min) are approximately equal to the rates of glucose removal by the tissues [9,72]. Glucose disposal is non-insulin-dependent and mostly concerns the brain (45–60%), but also red blood cells (5–10%), kidneys (10–15%), splanchnic tissues (3–6%), adipose tissue (2–4%), and skeletal muscle (15–20%) [72].

The liver is the predominant site for glucose production in the postabsorptive state and responsible for ~80% of the glucose released into the circulation, with the remaining ~20% contributed by the kidneys [73,74,75]. To support liver glucose production, most of the glucose utilized by peripheral tissues under fasting conditions is converted to lactate and alanine (which are cycled back to the liver) rather than oxidized [76,77,78]. Skeletal muscle is a major source of these substrates in the postabsorptive state and provides ~70% of the alanine and ~40% of the lactate released into the systemic circulation [78]. Muscle also releases glutamine which originates directly from proteolysis and accounts for ~45% of its plasma levels [79]. At normal concentrations of these amino acids in plasma, they make equivalent contributions to blood glucose. Plasma alanine levels are ~350 μM. If 70% of this comes from muscle, this is 245 μM and is sufficient to synthesize 125 μM glucose. Glutamine is present at ~550 μM in plasma; 45% of this comes from muscle, which is 247 μM, and this is also sufficient to synthesize 125 μM glucose. In support of these biochemical calculations, overall carbon transfer to the glucose pool from alanine and glutamine in postabsorptive humans is comparable (~3.47 and ~3.53 atoms/Kg/min, respectively) [80]. However, in contrast to glutamine, most of the alanine derived from muscle depends on glucose metabolism in this tissue and its conversion to pyruvate [49]. Perriello et al. [81] investigated the contributions of skeletal muscle proteolysis and plasma glucose to the production of these amino acids in postabsorptive humans and provided evidence for the existence of a “glucose-glutamine cycle” analogous to the “glucose-alanine cycle” and the “Cori cycle”. It was found that ~67% of the lactate and ~40% of the alanine but only ~13% of glutamine produced by muscle originated from plasma glucose. This means that ~33%, ~60%, and ~87% of the incorporation of lactate, alanine, and glutamine carbon into glucose is not immediately derived from plasma glucose [81]. Given the comparable overall amounts of plasma glucose derived from plasma glutamine and alanine, these results indicate that glutamine shuttles more non-glucose-derived carbon to the blood glucose pool than does alanine in the postabsorptive state; therefore, it is important for gluconeogenesis making use of the large protein reserves of skeletal muscle [80,81]. This is crucial when the flux of muscle proteins to support blood glucose levels is at its highest, such as during starvation [82,83,84].

**Figure 1:**
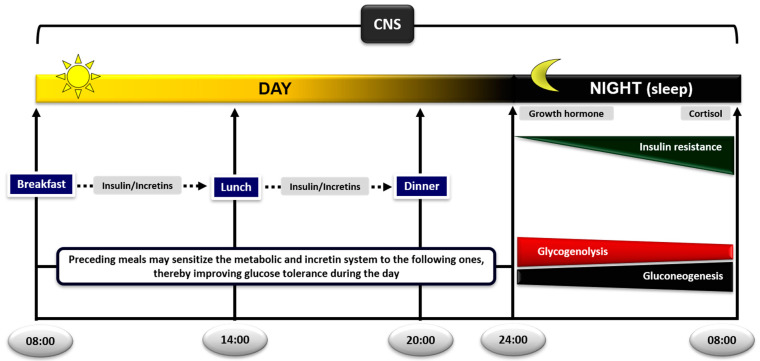
Diurnal regulation of glucose metabolism in the postprandial and postabsorptive state. The sequence of meals plays an important role in postprandial glycemic responses; preceding meals sensitize the metabolic and incretin system to the following ones, thereby improving glucose tolerance during the day. During sleep at night, the gradual development of insulin resistance, due to growth hormone and cortisol surges, ensures that blood glucose levels will be maintained within normal levels until awakening, by switching from glucose to nonesterified fatty acid (NEFA) oxidation in skeletal muscle. The increase in lipolysis and supply of NEFA to the liver and kidneys will also ensure stimulation of gluconeogenesis and glucose production (CNS: central nervous system).

A typical physiologic example of intermediate fasting is the overnight fast (e.g., 23:00–07:00). After the last meal of the day, the ratio insulin/glucagon increases initially to provide the stimulus for glycogen synthesis in the liver. Following the digestion of the meal and as fasting progresses during the night, the ratio insulin/glucagon decreases, and metabolism gradually moves from an anabolic into a catabolic state by the development of insulin resistance, which peaks at dawn [85,86,87] (Figure 1). The reason for insulin resistance is to redistribute glucose as a source of energy away from temporarily unessential skeletal muscle (the largest glucose-consuming tissue in the body) and direct it towards vital tissues which depend on the extracellular concentrations of glucose for survival (e.g., CNS and red blood cells) [88]). Under these conditions, muscle derives its energy-yielding fuels from noncarbohydrate substrates, such as NEFA [89]. With regard to the brain, a decrement in blood glucose levels as little as 20 mg/dL below normal fasting levels has been reported to decrease glucose uptake and trigger the release of counter-regulatory hormones [90]. Although the brain can also use ketone bodies as an alternative fuel for energy, their availability requires a number of metabolic changes in other tissues that could place a considerable burden on their function if glucose deprivation is prolonged [83,91].

The decrease in insulin/glucagon ratio firstly initiates the breakdown of liver glycogen. However, since the fasting liver almost completely depletes its glycogen stores within ~24 h, gluconeogenesis increases in parallel and gradually replaces glycogenolysis to support glucose production [92]. Indeed, under physiologic conditions of normal duration of fasting, about 50% of endogenous glucose production is due to glycogenolysis and the rest to gluconeogenesis in the liver and kidneys [93] (Figure 1). If fasting is prolonged, gluconeogenesis takes over and becomes the sole mechanism to support blood glucose levels [92,93]. These proportions may vary according to how much glycogen is stored in the liver in the beginning of fasting, which in turn depends on the amount of carbohydrate in the previous meal or on any performance of physical activity/exercise [92].

The development of insulin resistance in the early morning hours (usually around 04:00–06:00) is caused by the nocturnal surges of growth hormone and cortisol secretion governed by their circadian rhythm [85,94,95,96]. The pulse of growth hormone secretion occurs about an hour after the initiation of sleep due to changes in the activity of hypothalamus [94]. Since there is a few hours lag before growth hormone exerts its insulin-antagonistic effects [97], the resulting insulin resistance occurs in the early morning hours and coincides with the rise in cortisol levels at that time [98]. Elevation of growth hormone and cortisol induces insulin resistance in the liver and skeletal muscle [26,27,99,100] through a sequence of events: (a) HSL is activated and increases the rates of lipolysis and the production of glycerol and NEFA from the adipose tissue. (b) Increased plasma levels of NEFA decrease the rates of glucose uptake and oxidation in skeletal muscle. If fasting is prolonged, a parallel increase in the rates of proteolysis in this tissue increases amino acid production. (c) The supply of these substrates and NEFA to the liver and kidneys stimulate the rates of gluconeogenesis and glucose production [20,21,101,102,103,104]. Despite insulin resistance, blood glucose levels remain at constant physiological levels overnight by a modest and transient increase in insulin secretion just before dawn, which helps to restrain liver/kidney glucose production [85,86,87]. Experimental support for the significance of the nocturnal rise in growth hormone and cortisol to increase lipolysis at dawn has been provided by the group of Keith Frayn in Oxford. Overnight suppression of the growth hormone and cortisol surges in healthy subjects by somatostatin or metyrapone, respectively, decreased the activity of HSL in the subcutaneous adipose tissue, obliterating plasma NEFA increases in the early morning hours [105,106].

## 4. The Postprandial State

The magnitude of postprandial hyperglycemia depends on several factors including the sequence and composition of meals, the rates of gastric emptying and glucose absorption from the intestinal wall, the secretion and action of incretin hormones, insulin and glucagon, and the rates of glucose disposal by the liver and peripheral tissues (mainly skeletal muscle). These mechanisms are integrated via the central nervous system to regulate glucose intake, so it can match precisely to the energy needs of the tissues and avoid excessive hyperglycemia and hyperinsulinemia [107].

**Figure 2:**
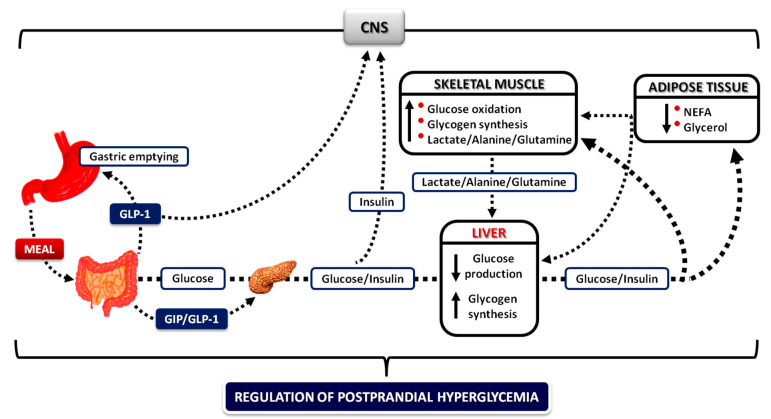
Integrative mechanisms for the regulation of postprandial hyperglycemia. During meal ingestion, several mechanisms operate in concert (gastric emptying and intestinal glucose absorption, secretion and action of gastrointestinal hormones, hyperglycemia mass action effects, and insulin/glucagon secretion and action) to ensure optimal regulation of postprandial glucose fluctuations in blood. Increased concentrations of insulin decrease adipose tissue lipolysis, resulting in a decrease in blood NEFA levels, which then mediate the decrease in endogenous glucose production permitting the initiation of glucose storage, and the increase in glucose uptake by skeletal muscle (CNS: central nervous system; NEFA: nonesterified fatty acids; GIP: glucose-dependent insulinotropic polypeptide; GLP-1: glucagon-like peptide-1).

After meals, the tissues must store and utilize the ingested glucose. This is achieved by an integrated interplay between food intake, gastric emptying/intestinal glucose absorption, gastrointestinal hormones, neural pathways, insulin/glucagon secretion, and action and glucose disposal. Increased concentrations of insulin in the postprandial period decrease adipose tissue lipolysis, resulting in a decrease in blood NEFA levels, which then: (a) mediate the decrease in endogenous glucose production and the increase in the rates of glucose utilization in skeletal muscle, and (b) help to incorporate the lipids of the meal into the adipose tissue triacylglycerol stores (Figure 2).

### 4.1. Oral Glucose Loads versus Mixed Meals

The data concerning tissues involved in the regulation of postprandial glucose homeostasis have been derived from studies in which glucose was administered either as a glucose load or as part of a mixed meal (liquid or solid) containing also fat and protein, using a variety of techniques [108]. McMahon et al. [109] compared the postprandial pattern of glucose metabolism in healthy subjects after an oral glucose load (50 g) or a solid mixed meal (451 kcal, 45% carbohydrate, 39% fat, and 16% protein); hepatic and extrahepatic glucose metabolism were assessed with the dual isotope and forearm catheterization techniques and indirect calorimetry. The results demonstrated that plasma glucose, insulin, and C-peptide levels as well as rates of systemic appearance of ingested glucose were all slightly higher during the first 15 min after the glucose load than after the mixed meal (due to a transient delay in gastric emptying and intestinal glucose absorption with the latter). However, despite these initial differences, after the first 15 min and until the end of the experiments (360 min) circulating glucose and hormone levels, rates of systemic entry of glucose, endogenous glucose production and muscle glucose uptake, glucose and lipid oxidation and nonoxidative glucose storage were virtually the same after the glucose drink and the mixed meal [109]. These findings agree with those of Jones et al. [110] showing that plasma glucose, insulin, and C-peptide levels were quite similar after administration of an oral glucose load (75 g) or a solid mixed meal (500 kcal, 60% carbohydrate, 20% fat, and 20% protein). Based on these results, McMahon et al. [109] concluded that insights regarding mechanisms of metabolic regulation of postprandial glucose fluxes derived from studies employing glucose loads are likely to pertain to those observed after mixed meals.

### 4.2. The Role of the Liver

After a mixed meal, the concentrations of glucose and amino acids rise in the portal vein and, through the liver, in the systemic circulation within less than 30 min (a longer interval is needed to produce rises in blood fat levels [111]). Nutrient absorption is followed by a rapid increase in the insulin/glucagon ratio, which, together with glucose, increases glucose uptake and storage by hepatocytes [112]. During its first pass through the liver, the secreted insulin is extracted by more than 60%; the rest enters the peripheral circulation and is cleared by the kidneys [113,114]. Therefore, the liver sees the largest changes in blood glucose and insulin concentrations after a meal and its role is to restrain their acute rise in the bloodstream [115] (Figure 2). The preferential use of the liver to dispose most of the ingested glucose protects the circulation from excessive hyperglycemia and hyperinsulinemia. This is of major physiological importance for two reasons: (a) acute or chronic hyperglycemia above an optimal level (>140–160 mg/dL) can induce oxidative stress and glycosylation of proteins disrupting cell metabolism [116,117,118,119]; it is also a prothrombotic factor [120]. (b) Hyperinsulinemia has growth-promoting effects [121], increases the risk for hypoglycemia leading to increased fluctuations of glucose [122], endothelial damage and proinflammatory/prothrombotic abnormalities [119,123,124,125], causes hypertension [126], and weight gain and can also promote insulin resistance [127,128].

The rates of glucose appearance in blood after a meal represent the sum of glucose escaping hepatic extraction after first pass and the residual release of endogenous glucose by the liver and kidneys [129]. The increase in posthepatic glucose delivery to the bloodstream is followed by a compensatory increase in glucose uptake by the peripheral tissues [130,131,132] (Figure 2).

Within 30–60 min after the beginning of a glucose load or a mixed meal and following rapid suppression of adipose tissue lipolysis and NEFA release by insulin [53,89,133], endogenous glucose production is suppressed almost completely, and the liver directly incorporates an average of 50% of the ingested glucose into glycogen (this percentage varies between publications from ~40% to ~70% due to differences in experimental conditions and techniques) [134,135,136,137,138,139]. The remaining of the ingested glucose passes through the liver and enters the systemic circulation to be used by peripheral tissues as follows (estimated values): ~26% is taken up by skeletal muscle, ~23% by the brain, ~3% by the adipose tissue, ~7% by the kidneys, and the remaining by other tissues (heart, erythrocytes, skin, etc.) [135] (Figure 2). Therefore, liver, muscle, and brain appear to be the major sites of glucose disposal, together accounting for ~80% of the load. Support for the significance of the decrease in plasma NEFA levels for postprandial glucose assimilation by the liver has been provided by Kruszynska et al. [140]: infusions of a triglyceride emulsion to prevent circulating plasma NEFA levels from falling after a glucose load impaired insulin’s ability to suppress hepatic glucose production.

### 4.3. The Role of Skeletal Muscle and Adipose Tissue

**Figure 3:**
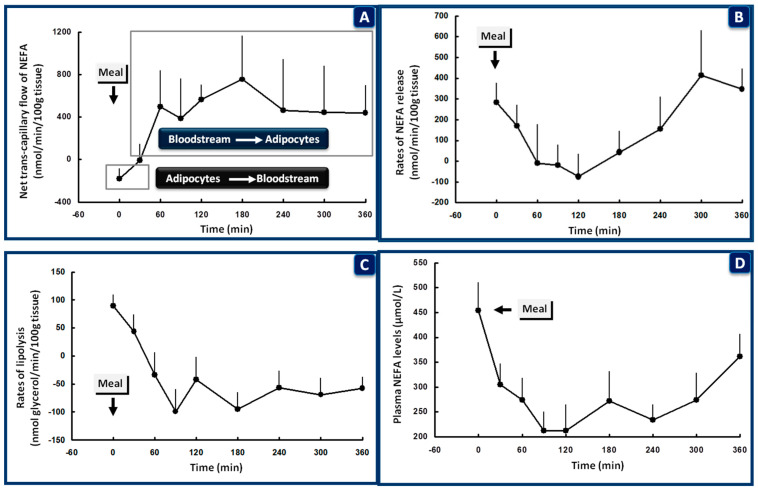
Nonesterified fatty acid (NEFA) kinetics in the subcutaneous adipose tissue of healthy subjects after a mixed meal given at 08:00, following overnight fasting. At the beginning of the meal (0 time), the trans-capillary flow of NEFA is from the adipocytes to the bloodstream (**A**). The rates of NEFA release (**B**), lipolysis (**C**), and plasma levels of NEFA (**D**) are all increased to cover energy requirements in muscle during sleep and sustain endogenous glucose production. Within 30 min after the beginning of the meal and in the presence of increasing blood levels of insulin, trans-capillary flow of NEFA is reversed from the bloodstream to the adipocytes (**A**). The rates of NEFA release (**B**), lipolysis (**C**), and plasma levels of NEFA (**D**) are all rapidly suppressed to allow: (a) storage of the ingested lipids in the adipose tissue; (b) suppression of endogenous glucose production by the liver and kidneys and initiation of glucose storage in the liver; (c) stimulation of glucose uptake in skeletal muscle (data from [149]).

In muscle, glucose extraction starts within 15–30 min and reaches a peak at ~60–90 min after a glucose load, in synchrony with the peak of plasma insulin levels [130,135,141]. The increase in insulin-stimulated blood flow also contributes to the glucose extraction in this tissue [64]. Following uptake, glucose is initially oxidized to replace NEFA oxidation used as an energy source during the precedent fast [135]. The quick (within less than 30 min) switch from fat to glucose oxidation in the fasting to postprandial transition (and vice versa) [142,143] is the cornerstone of metabolic regulation and has been defined by Kelley and Mandarino as “metabolic flexibility” [144,145,146] (Figure 3).

In support of these findings, the experiments by Taylor et al. [147] using ^13^C-nuclear magnetic resonance spectroscopy to measure glycogen synthesis in the gastrocnemius muscle after a solid mixed meal showed that net glycogen synthesis started at 90 min after the beginning of the meal, when the rates of glucose uptake were markedly increased, and the increase was not significant until 3 h after eating. Therefore, from these studies, it can be inferred that the predominant fate of glucose taken up by muscle in the immediate postprandial period is oxidation [135,147]. Saturation of the glucose oxidation pathway during the latter postprandial period by the increased plasma concentrations of insulin will be followed by net glycogen synthesis [147,148].

When muscle glycogen stores are replete, if the rates of glucose uptake remain elevated, glucose will be converted to lactate, alanine, and glutamine; these substrates will be directed to the liver to incorporate their carbons into glycogen (indirect pathway of glycogen synthesis) [112,129,147] (Figure 2). Not only do they allow glucose to be converted to gluconeogenic precursors when muscle glycogen stores are replete, but they may also function as a “temporal buffer” for glucose [2]. Thus, conversion to lactate and alanine (circulation in the blood and then uptake and conversion to glycogen in the liver) could prevent abrupt fluctuations and help maintain a constant blood level of glucose after ingestion of carbohydrates. Therefore, considering both the direct (first pass) and indirect pathways of glycogen synthesis from 3-carbon fragments derived from glucose, overall, the liver disposes more than 50% of the ingested glucose after a meal [150], allowing a much smaller amount of the glucose load to remain in the systemic circulation and be removed by peripheral tissues [135]. However, if the liver is overpowered by increased amounts of carbohydrate in the meals, the spillover of glucose to the peripheral circulation will be much higher engaging the muscle for its removal; obviously, this will require a substantial increase in systemic insulin levels and insulin secretion. In the long term, this mechanism provides an insight into how a high (in contrast to a low) glycemic index/load carbohydrate diet producing sustained high insulin secretion could contribute to the development of insulin resistance [151,152,153,154].

The interplay between liver and muscle in postprandial glucose handling has recently been examined in the study of Kowalski et al. [155]. The administration of three different glucose loads (25, 50, or 75 g) to healthy subjects combined with the dual-tracer technique to measure hepatic glucose production showed similar plasma glucose responses for all doses tested; however, there was a dose-dependent increase in insulin secretion and plasma insulin levels (as discussed by the authors this was probably due to incretin-mediated effects). Although hepatic glucose production was rapidly and equally suppressed across all doses, there was a dose-dependent increase in the rates of glucose disposal by peripheral tissues induced by the progressive hyperinsulinemia, which accounted for the lack of the dose effect on postprandial plasma glucose increases. The authors [155] concluded that: (a) the body preferentially relies on the liver for a disposition of small glucose loads since this requires minimal stimulation of insulin secretion, and (b) muscle may represent a backup mechanism that relies on hyperinsulinemia and is engaged only when the glucoregulatory effects on the liver reach capacity. These conclusions are supported by the findings of Rizza et al. [9] showing that the liver is much more sensitive to insulin than skeletal muscle: (a) the concentrations of insulin causing half-maximal suppression of glucose production (~29 μU/mL) are considerably lower than those required for half-maximal increase of glucose utilization by muscle (~55 μU/mL) [9], and (b) glucose production is completely suppressed at plasma insulin concentrations of ~60 μU/mL (normally seen after meals), whereas maximal glucose utilization by muscle occurs at insulin concentrations above 200 μU/mL [9]. Adipose tissue, a key metabolic coordinator of liver–muscle interplay, is also extremely sensitive to insulin: the half-maximal suppression of lipolysis (measured by the rates of glycerol release) occurs at plasma concentrations of insulin as low as 13 μU/mL [156].

These results, taken together with the inhibition of glucagon secretion by insulin within 30 min after the beginning of the meal, the simultaneous inhibition of hepatic glucose production, the efficient incorporation of a large part of glucose into glycogen during first-pass through the liver, the high clearance of the secreted insulin by the liver, the quick suppression of adipose tissue lipolysis and NEFA/glycerol release with even tiny increases in the peripheral concentrations of insulin, and the liver–muscle interplay (summarized in reference [108]) (Figure 2 and Figure 3), support the suggestion of Kowalski et al. [155], that the liver may represent an evolutionary conserved mechanism for the regulation of postprandial hyperglycemia, with a mission to reduce the secretory burden on β-cells and therefore avoid the negative consequences of hyperinsulinemia.

### 4.4. The Role of de Novo Lipogenesis

Following repletion of glycogen stores, excess carbohydrate can also be disposed by glucose conversion into lipids (fatty acids, and hence triacylglycerol), a function known as de novo lipogenesis [157]. Studies with human tissues have shown that the key enzymes for fatty acid synthesis from glucose are present not only in the liver and adipose tissue [158], but also in skeletal muscle [159]. We will report the results of five studies that examined the role of de novo lipogenesis in postprandial glucose handling: (a) Acheson et al. [160] gave a 500-g carbohydrate meal to healthy subjects and measured whole-body net fat synthesis by using indirect calorimetry. The results showed that, for 10 h following the meal, fat synthesis from glucose was low and did not exceed fat oxidation. (b) The same authors [161] studied de novo lipogenesis for 24 h after ingestion of 500-g of carbohydrate in subjects who had consumed a high carbohydrate diet (80% carbohydrate, 9% fat, and 11% protein) for 3–6 days preceding the test. Under these conditions, a small amount of net lipogenesis did occur for a few hours postprandially, but there was no net gain of body fat from glucose over the whole 24-hour period [161]. (c) Aarsland et al. [162] gave a liquid carbohydrate-rich meal (83% carbohydrate, 15% amino acids, and 2% fat) to healthy subjects through a feeding tube placed into the upper gastrointestinal tract, before and after 1 and 4 days on a hyperenergetic carbohydrate diet (2.5 times more than their daily energy requirements). Whole-body net fat synthesis and substrate exchange were measured by indirect calorimetry and intravenous tracer infusions. Although there was no net whole-body fat synthesis in the basal experiment, this was increased after 1 and particularly after 4 days of carbohydrate overfeeding. The main site for fat synthesis from glucose was shown to be the adipose tissue rather than the liver [162]. (d) Aas et al. [159] examined the effects of hyperglycemia per se on lipid synthesis in primary human muscle cells incubated in the presence of high (360 mg/dL) or physiological concentrations of glucose (99 mg/dL) for 24 h. In the presence of hyperglycemia, the conversion of glucose to NEFA and triacylglycerol was markedly increased by ~88% and ~44%, respectively. In the presence of euglycemia, de novo lipogenesis was negligible (less than 5%) [159]. (e) In the study by Solinas et al. [163], incubation of rat muscle preparations in buffer containing [^14^C]-labeled glucose resulted in the production of [^14^C]-labeled NEFA and triacylglycerol demonstrating that de novo lipogenesis can indeed occur in intact skeletal muscle and can be stimulated by insulin, as in the liver and adipose tissue. However, given the well-known connection between lipid accumulation in skeletal muscle and insulin resistance [164], the physiological significance of de novo lipogenesis in this tissue may not be obvious. This was clarified by the same group of authors [165] who provided evidence for the existence of a substrate cycle between de novo lipogenesis and lipid oxidation in incubated rat muscle, which may enable lipids derived from glucose to be subsequently oxidized, and, therefore, not accumulated. This substrate cycle was shown to be regulated by an interaction between insulin (which increases lipid synthesis) and leptin (which increases lipid oxidation) [163,165,166]. Although leptin is believed to be involved in the long-term maintenance of energy balance, its diurnal rhythm has been shown to be entrained to meal timing [167]. The existence of a substrate cycle would be expected to increase the sensitivity of this metabolic pathway to insulin and leptin, and hence increase its flexibility in responding to excess carbohydrate consumption [168].

Taken together, these results suggest that, under conditions of regular mixed meal consumption de novo lipogenesis does not play a major role and postprandial glucose fluctuations are handled successfully by glycogen storage and glucose oxidation. However, under conditions of carbohydrate-rich diets with high glycemic index/load foods that overwhelm glycogen stores, de novo lipogenesis may contribute to the postprandial glucose disposal in the liver, adipose tissue, and skeletal muscle, leading, however, to an increase in their fat reserves.

### 4.5. The Role of the Kidneys

The contribution of the kidneys to the assimilation of postprandial glucose should also be discussed. In the fasting state, the kidneys use NEFA as the major oxidative fuel [169]. However, in agreement with previous findings [135], experiments by Meyer et al. [129] have shown that in the postprandial state, the kidneys account for as much as ~10% of the disposal of a 75-g glucose load. The uptake of glucose is stimulated by hyperinsulinemia, hyperglycemia (mass effect), and the increases in blood flow rates; glucose is anaerobically metabolized in the kidneys and released as lactate [129,169]. The substitution of glucose for NEFA oxidation under conditions of hyperglycemia provided evidence for a “glucose-fatty acid cycle” in the kidneys, analogous to that observed in skeletal muscle [170]. Meyer et al. [129] also showed that, in contrast to what would be expected, kidney gluconeogenesis increases by about twofold after a meal towards the end of the digestion period. As it was suggested by these authors [129], this increased postprandial release of glucose by the kidneys would permit greater suppression of hepatic glycogenolysis so that there would be more efficient refill of the glycogen stores in the postprandial period, a function that the kidneys do not have. 

### 4.6. The Role of the Gastrointestinal Tract

The importance of the gastrointestinal tract in postprandial glucose metabolism has been the focus of much attention for over 60 years. Early studies had shown that oral glucose administration results in a greater insulin secretory response than that observed with intravenous glucose, suggesting that the intestinal absorption of glucose can stimulate insulin release by a mechanism independent of changes in blood glucose levels [171,172]. Perley and Kipnis [173] examined the contribution of the insulinogenic stimuli arising from the gastrointestinal system or from the circulation (e.g., plasma glucose levels) on β-cell secretion, after oral or intravenous administration of glucose to reproduce the blood glucose levels achieved by the oral load. They showed that, despite similar increases in plasma glucose levels, oral glucose ingestion resulted in higher plasma insulin responses and higher (over 60%) retention of the glucose load in the liver after oral vs. intravenous administration, thus suggesting that an alimentary signal in the upper gastrointestinal tract had been involved [173]. In a series of experiments combining glucose-insulin clamps, oral administration of glucose and catheterizations of brachial artery and hepatic vein, DeFronzo et al. [174] examined the relative contributions of hyperinsulinemia, hyperglycemia (alone or in combination) and the route of glucose administration on liver glucose disposal. The results indicated that hyperinsulinemia or hyperglycemia induced by intravenous infusion of glucose or insulin caused minimal net uptake of glucose by the liver (~4%–14%) despite marked stimulation of total glucose turnover. In contrast, after oral administration of glucose net liver uptake increased to values sixfold higher than those with intravenous glucose. These authors [174] concluded that orally consumed glucose caused a release of a gastrointestinal factor that enhanced insulin-mediated uptake of glucose by the liver. These results were subsequently supported by the findings of Ishida et al. [175] in conscious dogs: hepatic glucose uptake after administration of equal amounts of glucose orally or by intraportal or intravenous infusions was 68%, 65%, and 23%, respectively. Further details on the relevant hormones will be discussed in the following sections.

#### 4.6.1. Gastric Emptying

The rates of gastric emptying and intestinal glucose absorption determine the feedback relationship between the increases in blood glucose levels and insulin secretion, and therefore play a decisive role in the magnitude of postprandial hyperglycemia and hyperinsulinemia [176]. Following an oral glucose load, gastric emptying has also been shown to be associated with decreased hunger and increased fullness and satiety [177,178].

Gastric emptying is regulated by gastric and intestinal signals arising from the interaction of nutrients with the gastrointestinal wall (which play a dominant role) by the levels of glucose in the blood and by neural feedbacks [107,179]. More specifically, hyperglycemia within the physiological range decreases [180], whereas low blood glucose levels accelerate gastric emptying after solid or liquid meals [181,182]. Ghrelin, a hormone secreted from the stomach accelerates [183], whereas hormones secreted from the proximal (secretin, cholecystokinin, and somatostatin) [184,185] and the distal small intestine (glucagon-like peptide-1/GLP-1 and peptide YY) inhibit gastric emptying [186,187].

Since carbohydrates are absorbed in the proximal small intestine, the entry of glucose into the portal vein and its appearance to the liver and the peripheral tissues after a meal will be influenced mainly by the rate of delivery from the stomach [188]; this was recognized as early as 1915 [189]. The rates of gastric emptying have been shown to correlate positively with postprandial hyperglycemia and may account for over 30% of the variance in plasma glucose levels after oral glucose loads or solid meals [190,191,192]. More specifically, the increases in blood glucose levels between 0–30 min after a 75-g oral glucose load (responsible for the acute/first phase insulin secretion from the β-cells) were shown to be directly related to gastric emptying; however, the blood glucose levels at 120 min were inversely related to gastric emptying, obviously due to the early surges of insulin release [193,194].

The importance of the rate of glucose delivery from the stomach into the intestinal tract as a major determinant of the glycemic and insulin responses has been investigated by Jenkins et al. [195] by administering 50-g glucose loads to healthy subjects on two occasions: over 5–10 min (bolus) or as a constant rate over 3.5 h. Despite similar blood glucose areas over the whole experimental period, plasma insulin and C-peptide levels were about 50% lower, plasma NEFA levels were more effectively suppressed and plasma glucose-dependent insulinotropic polypeptide (GIP) levels were 40% lower when glucose was given at a constant rate compared to when it was administered as a bolus. These authors [195] concluded that slower rates of glucose delivery into the small intestine and gradual absorption of the ingested glucose enhance insulin economy and glucose disposal and improve tissue sensitivity to insulin. In line with these observations, the rate of eating can substantially affect postprandial glycemic and insulin responses [196]. Eating quickly is followed by a faster entrance of glucose into the circulation, requiring an immediate and therefore higher insulin secretion from the β-cells. Indeed, eating fast can induce insulin resistance by the chronic elevations of plasma insulin levels [127,197]. In contrast, eating at a slower rate is associated with lower energy intake, increased satiety, higher plasma levels of the anorexigenic peptides GLP-1 and peptide YY, and lower levels of the orexigenic peptide ghrelin [198,199].

#### 4.6.2. Gastrointestinal Hormones

In response to food and via feedback mechanisms, tightly coordinated by the central nervous system, the gastrointestinal tract secretes several peptides that regulate its motor function and absorption capacity, but also hunger, satiety, fullness, and insulin/glucagon secretion [200,201].

##### Ghrelin

Ghrelin is predominantly produced by the neuroendocrine cells of the stomach [202,203] but is also detected in many other endocrine/nonendocrine peripheral tissues, as well as in the hypothalamus and the brainstem [204]. Although it was discovered as a factor increasing growth hormone secretion via central effects, subsequent findings provided evidence that ghrelin plays an important role in food intake, energy balance, and metabolic control as an endocrine link between stomach, hypothalamus, and pituitary [205]. Administration of ghrelin to rodents or humans increases appetite, food intake, adiposity, and body weight, and reduces fat utilization [205,206].

Ghrelin is secreted just before an anticipated meal to induce a prompt increase in gastric motility at the beginning of the meal and declines within less than one hour after the beginning of the meal, as the blood concentrations of intestinal hormones rise [207,208]. Early studies showed that ghrelin stimulates insulin secretion in the presence of hyperglycemia [209]. On the other hand, the increase in plasma insulin levels after a meal was shown to be a prerequisite (along with hyperglycemia and low plasma NEFA levels) for the postprandial suppression of ghrelin [210]. These observations suggest a competitive interaction between ghrelin, insulin, and key substrates in the short-term regulation of postprandial glucose metabolism at the beginning of the meal.

Ghrelin has receptors in the arcuate nucleus of the hypothalamus along with insulin and leptin [211]. Leptin is a hormone involved primarily in long-term regulation of energy balance that serves as a measure of energy reserves, directing the central nervous system to adjust food intake and energy expenditure accordingly [212]. Whereas ghrelin has an orexigenic effect signaling a state of hunger from the stomach, both insulin and leptin have anorexigenic effects, signaling states of carbohydrate and lipid abundance, respectively [213,214,215]. The orexigenic effects of ghrelin are mediated by neuropeptide Y (NPY), a potent appetite stimulator [216]. Whereas the downstream signaling from the leptin receptor inhibits the neural pathway in response to the NPY, ghrelin relieves this inhibition [217]. For these reasons, the ratio of leptin to ghrelin in the bloodstream has been suggested to be an important determinant of appetite, food intake, and energy balance [215]. However, insulin and leptin have synergistic effects at the hypothalamus, indicating that these hormones both operate to bring about an anorexigenic effect, but by the utilization of separate signaling pathways [218,219]. These roles are augmented by the control of each hormone’s secretion by the other: insulin stimulates leptin production from the white adipocytes [220] while leptin suppresses insulin release from the pancreatic β-cells [221]. The central and peripheral actions of ghrelin, insulin, and leptin are thus coordinated to ensure that appropriate behavioral responses follow from carbohydrate and fat repletion.

Following synthesis, ghrelin is acylated with an ester-linked fatty acid group to an active form, and then cleaved to a nonacylated form in blood and peripheral/central tissues by several esterases [222,223,224]. Circulating total ghrelin is therefore the sum of the acylated and nonacylated forms, with the concentrations of the latter in blood representing 95% and the former less than 5% of the total [225]. It should be noted that early studies investigating ghrelin action obviously referred to total ghrelin (acylated plus nonacylated forms), and therefore the effects reported were an integration of the actions of both peptides. Below, we outline some more recent studies dissecting the effects of both forms of ghrelin.

Although nonacylated ghrelin was initially thought to be a degradation product of ghrelin and therefore inactive [202], studies over the past few years have shown that it has pleiotropic actions and can antagonize the metabolic but not the endocrine effects of acylated ghrelin [226]. It has therefore been suggested that acylated and nonacylated ghrelin should be considered as different hormones regulated by separate mechanisms. The evidence is as follows: (a) By using specific assays, Liu et al. [227] showed that under normal everyday conditions, acylated and nonacylated ghrelin were both increased just before the initiation of meals, were sharply inhibited postprandially and increased at night. However, during long-term fasting (60 h) when blood levels were relatively low, acylated ghrelin decreased over 60% to levels seen postprandially, but the nonacylated form remained to its peak levels seen preprandially. These authors [227] suggested that acylation may be regulated independently of secretion, by nutrient availability in the gut, by esterases that cleave the acyl-group, or both. (b) In mice and rats, administration of acylated ghrelin stimulated food intake by activating orexigenic peptides in the hypothalamus and enhanced gastric emptying, whereas nonacylated ghrelin had anorexigenic effects and decreased gastric emptying [228,229]. (c) Gauna et al. [230] examined the effects of acute administration of acylated ghrelin, nonacylated ghrelin, and their combination on insulin sensitivity in growth hormone-deficient patients after a mixed meal. Intravenous injections of acylated ghrelin decreased insulin sensitivity and increased lipolysis and serum NEFA levels, whereas the coadministration of nonacylated ghrelin improved insulin sensitivity and suppressed lipolysis and serum NEFA levels for up to 6 h. As suggested by these authors [230], the ratio of acylated/nonacylated ghrelin may therefore be involved in the regulation of the balance between adipogenesis and lipolysis in response to the nutritional status. Similar results were reported by Vestergaard et al. [231] using hyperinsulinemic-euglycemic clamps and infusions of labeled glucose to measure glucose turnovers. Continuous infusions of acylated ghrelin in healthy subjects and hypopituitary patients for 300 min acutely induced insulin resistance, increased lipolysis, and serum NEFA levels and suppressed insulin-stimulated glucose disposal [231]. (d) Incubation of primary hepatocytes with acylated and nonacylated ghrelin showed that the former stimulates, whereas the latter inhibits glucose output even in the presence of glucagon [232]. (e) Fasting acylated/nonacylated ghrelin ratios have been found high in insulin resistant conditions (such as the metabolic syndrome) and low in insulin sensitive conditions [233,234]. (f) In healthy subjects, infusions of acylated-ghrelin to achieve increases within the physiological range, did not affect fasting insulin and glucose levels but restrained first-phase insulin secretion and the elevations of plasma insulin concentrations after an intravenous glucose load; at the high dose, acylated ghrelin also deteriorated glucose tolerance [235]. In contrast, nonacylated ghrelin had opposite effects and increased glucose-stimulated insulin secretion [236]. Interestingly, Page et al. [237] recently showed that administration of acylated ghrelin during meal consumption increased GLP-1 secretion, a well-known stimulator of glucose-induced insulin release. These results imply that the secretion of GLP-1 has a role to counterbalance the effects of acylated ghrelin to suppress insulin secretion and deteriorate glucose tolerance [237]. (g) Spiegel et al. [238] examined the 24-hour fluctuations of plasma acylated and total ghrelin (which mainly reflects the nonacylated form) levels in healthy subjects given three identical mixed meals (60% carbohydrates, 30% fat, and 10% protein) at 5-hour intervals. The fluctuations of acylated/total (nonacylated) ghrelin were independent of the time of day and were related only to meal consumption. The ratio acylated/total (nonacylated) ghrelin was higher at the beginning of meals and lower during sleep at night, consistent with an increase and a decrease of the orexigenic signals, respectively. There was a strong positive association between the 24-hour fluctuations of acylated ghrelin and the postprandial glucose levels; the individuals with higher plasma acylated ghrelin levels had also higher and more prolonged glucose responses to the carbohydrate-rich meals [238]. In agreement with previous suggestions [230], the results of Spiegel et al. [238] support the distinct interaction of acylated and nonacylated forms of ghrelin in the regulation of glucose homeostasis under fed or fasted conditions. (h) In a recent study, Togliato et al. [239] demonstrated that nonacylated ghrelin has also protective effects on muscle endothelial cells subjected to ischemia by activating endothelial oxidative defense, therefore playing a role in the prevention of cellular damage.

Taken together, these observations indicate that ghrelin is primarily involved in the storage of ingested calories, as initially proposed [205]: it is released into the circulation as a function of energy stores and its changes in the blood are clearly meal related. The postprandial decreases of ghrelin limit the intake of additional food if energy requirements have been fulfilled. The ratio of acylated/nonacylated ghrelin (in collaboration with the intestinal hormones) induces a state of positive or negative energy balance, respectively, to adjust the overall handling of glucose to the occasional metabolic needs.

##### Intestinal Hormones

The incretins are gut peptides secreted within minutes in response to food ingestion and play a crucial role in the regulation of insulin/glucagon secretion and glucose homeostasis [201]. The two major incretins that regulate insulin and glucagon release are glucagon-like peptide-1 (GLP-1) and glucose-dependent insulinotropic polypeptide (GIP) [240,241]. In response to glucose absorption from the intestinal wall, GLP-1 is released from the L-cells of the distal ileum and colon, whereas GIP is secreted by the K-cells located predominantly in the duodenum and proximal jejunum [242,243]. Both peptides have receptors on the pancreatic β-cells and α-cells and generate signals that stimulate glucose uptake [244,245,246,247].

GLP-1 has been extensively studied due to its wide application in the treatment of type 2 diabetes [248,249] and has several actions that control meal-related glycemic excursions. Since the location of the L-cells is mostly in the distal and not the proximal small intestine (glucose rarely reaches the distal ileum unless the load ingested is too high), the rapid increase of GLP-1 levels in blood after food ingestion is due not only to the presence of nutrients in the gut but also to central mechanisms via the hypothalamus and brainstem [107]. GLP-1: (a) increases satiety, promotes fullness, and decreases hunger; these effects reduce food intake and promote weight loss [250]. (b) It retards the motility of the stomach and small bowel, slows gastric emptying, and delays intestinal glucose absorption [251]. (c) It potentiates glucose-dependent insulin secretion from the β-cells and inhibits glucagon secretion from the α-cells in the presence of hyperglycemia (increases insulin/glucagon ratio) [252]. GLP-1 augments both the first and second phase of insulin response to glucose, with the most predominant effect on the first (acute) phase [253]. (d) The combined effects of GLP-1 to inhibit glucagon secretion, delay gastric emptying, and reduce food intake and body weight [201] are expected to increase insulin sensitivity indirectly, and hence facilitate a reduction in hepatic glucose production and an increase in muscle glucose disposal after meals. The effects of GLP-1 on endogenous glucose production and peripheral glucose uptake have been examined by three studies. Larsson et al. [254] gave GLP-1 as a continuous infusion for 180 min in fasting individuals without or with somatostatin to inhibit insulin and glucagon secretion, along with labeled glucose to measure glucose turnovers. GLP-1 alone increased the ratio insulin/glucagon as expected, resulting in a decrease in plasma glucose levels and in endogenous glucose production and a parallel increase in the metabolic clearance rate of glucose. The concomitant infusion of somatostatin abolished the effects of GLP-1 on glucose turnovers, leaving plasma glucose levels unaffected. These authors [254] concluded that the effects of GLP-1 on the liver and peripheral tissues are mediated indirectly by the changes in insulin/glucagon ratio. In a more recent study, Seghieri et al. [255] gave GLP-1 as a continuous intravenous infusion for 4 h to mimic physiological postprandial levels. The secretion of insulin and glucagon was suppressed by infusion of somatostatin (both hormones were replaced at basal levels); labeled glucose and glycerol were also infused to measure glucose and lipid turnovers. Under these experimental conditions plasma glucose levels gradually rose to a plateau of ~190 mg/dL and endogenous glucose production increased by ~60%. During GLP-1 infusion at matched plasma glucose levels, the rise of glucose production was fully prevented, and peripheral glucose disposal and lipid kinetics were unaffected. These results provide convincing evidence that GLP-1 inhibits endogenous glucose production under conditions where its major controlling signals are not allowed to change. As suggested by these authors [255], this effect may be either direct on the hepatocytes or via neural stimuli following meal ingestion. Finally, D’Alessio et al. [256], by using the intravenous glucose tolerance test and the minimal model of glucose kinetics to derive indices of insulin sensitivity and glucose effectiveness, suggested that GLP-1 may have direct effects on tissues involved in postprandial glucose handling by potentiating insulin-independent glucose disposal (i.e., induced by hyperglycemia per se) in the liver and/or skeletal muscle.

GIP has many similarities with but also differences from GLP-1 [257]. GIP, like GLP-1, enhances glucose-induced insulin secretion and β-cell proliferation; the effects of the two peptides on β-cells are additive [258]. However, in contrast to GLP-1, GIP does not delay gastric emptying after meal ingestion and has no important role in satiation signaling [259,260,261]. Moreover, in contrast to GLP-1, GIP has a glucagonotropic action in humans, which, however, depends on the prevailing glucose concentrations: glucagon secretion is increased by GIP in the presence of euglycemia or hypoglycemia but not in the presence of hyperglycemia [262]. Therefore, as suggested by Christensen et al. [263] GIP has a physiological bifunctional role as a blood glucose stabilizer, with diverging glucose-dependent effects on the two main pancreatic glucoregulatory hormones, insulin and glucagon.

The timing of GLP-1/GIP secretion and their interplay with insulin release after nutrient ingestion deserves some discussion. In an elegant series of experiments, Schirra et al. [264] gave 50 or 100 g of glucose orally or directly into the duodenum as an infusion and investigated the relationship between gastric emptying/duodenal perfusion of glucose and the release of GLP-1, GIP, and insulin. The results of this important study showed that: (a) GLP-1 and GIP were independently and highly associated with insulin release during the first postprandial hour, supporting their crucial role as gastrointestinal determinants of postprandial insulin release. (b) Oral ingestion of glucose markedly increased plasma levels of both GLP-1 and GIP. However, intraduodenal infusion of glucose increased GIP but not GLP-1 release supporting the evidence that, in contrast to GLP-1, GIP is not causally related to gastric emptying but is governed solely by the presence of glucose in the intestinal lumen. (c) GIP release was independent of the amount of glucose administered. In contrast, GLP-1 showed a threshold of caloric delivery from the stomach into the duodenum, which had to be exceeded to stimulate measurable release into the circulation. Therefore, with regards to incretin effects, glucose-stimulated insulin secretion after ingestion of low glycemic index/load foods may be mediated mainly by GIP not GLP-1. (d) Postprandial plasma insulin concentrations increased to peak values at 30–45 min and had declined to basal levels by 180 min with both glucose loads. Plasma GLP-1 and GIP levels peaked at 20–30 min with both loads postprandially. Thereafter, GLP-1 sharply declined, with basal values reached at 60 or 120 min after the 50 and 100 g glucose loads, respectively. Interestingly, plasma GIP levels did not decline but remained elevated close to the peak levels over the whole experimental period, although plasma glucose and insulin levels had returned to baseline [264]. Considering the glucagon-stimulating effects of GIP at low but not high plasma glucose levels [262], the results by Schirra et al. [264] suggest that this may be a mechanism to protect from postprandial hypoglycemia (transient rebound of glucose levels), a frequent finding after the ingestion of high glycemic index/load nutrients, due to postprandial hyperinsulinemia.

These findings, taken together, suggest that there are three interacting events that precede insulin secretion after a meal: the release of GIP, the release of GLP-1, and the entry of glucose into the circulation following intestinal absorption. The coordinated interplay between these events helps to adjust the amount of ingested glucose to the metabolic needs in the postprandial state, thus helping to avoid not only excessive hyperglycemia and hyperinsulinemia but also postprandial hypoglycemia.

### 4.7. The Biphasic Manner of Insulin Secretion

The pancreatic β-cells couple the metabolism of carbohydrates, proteins, and lipids to the insulin secretory mechanisms, such that insulin secretion occurs at appropriate amount and timing to ensure efficient nutrient uptake and storage by target tissues [265,266]. The β-cells act as a glucose sensor matching its output to the glucose levels in the bloodstream via metabolically induced changes in electrical activity; this leads to increases in cytoplasmic Ca^2+^ concentrations and initiation of Ca^2+^-dependent exocytosis of insulin-containing secretory granules [267]. Although glucose is the major physiological stimulus for insulin release, amino acids and fatty acids can also elicit insulin secretion [265,268]. However, as reported by Rorsman and Ashcroft [267] from experiments in rodent and human islets, the primary signal for insulin secretion is not usually glucose but the neurotransmitters and incretins secreted just before or at the beginning of the meal. As suggested by these authors [267], the important function of this is to prepare the body for the subsequent increase in blood glucose levels and therefore prevent blood glucose from rising too high in the postprandial period.

**Figure 4:**
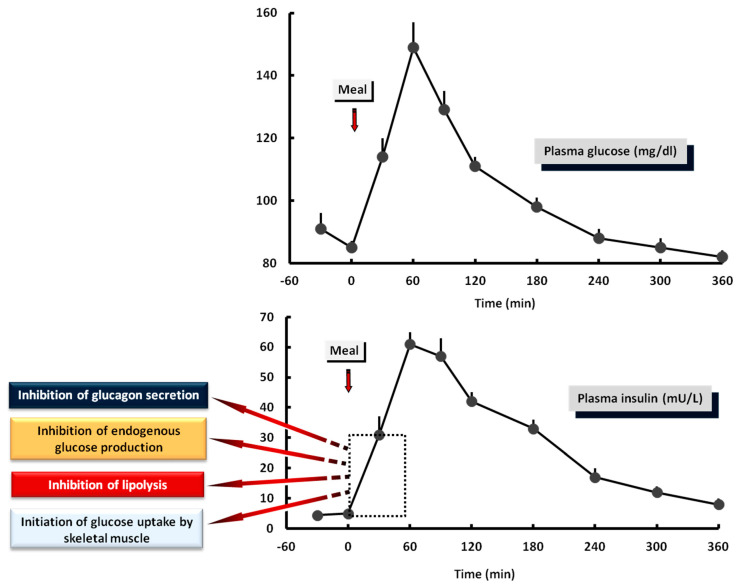
Plasma levels of glucose and insulin after a solid mixed meal in healthy subjects. The early response of insulin: (a) inhibits glucagon secretion from the pancreatic α-cells. (b) Inhibits lipolysis in the adipose tissue. (c) Suppresses endogenous glucose production. (d) Initiates glucose uptake from skeletal muscle. These preliminary effects of insulin start within the first 30 min after the beginning of the meal and are critical for optimal glucose regulation in the postprandial period.

The increase in insulin secretion by the β-cells after glucose stimulation occurs in a biphasic manner: a rapid marked release (first phase) and a more sustained and less elevated release (second phase) [269,270]. The early phase of insulin secretion is critical for glucose regulation in the postprandial state for the following reasons: (a) via paracrine effects, induces a rapid inhibition of glucagon secretion from the pancreatic α-cells at the beginning of the meal. The increase in insulin/glucagon ratio switches off the stimulatory effects of glucagon on glucose production by the liver, thus facilitating the suppressive effects of insulin. The following second phase is important to sustain the inhibition of glucagon and augment glucose storage and utilization [271]. (b) Within 30 min after the beginning of the meal, it induces a prompt suppression of lipolysis by inactivating HSL in the adipose tissue, thus decreasing blood NEFA and glycerol levels [53,89,156,272]. The decrease in NEFA levels is of major importance since it mediates the suppression of endogenous glucose production in the liver/kidney and the increase in the rates of glucose utilization in muscle [20,21]. (c) It is essential for the activation of LPL in the adipose tissue and the clearance of triacylglycerol from the bloodstream after the meal [273] (Figure 4).

### 4.8. The Role of Hyperglycemia

Glucose can regulate its own metabolism in the tissues independently of insulin [10,274,275,276]. Hyperglycemia per se stimulates glucose uptake in the liver and skeletal muscle by increasing the activity of its transporters on the surface cell membranes (mass action effect) [277,278,279]. However, acute rises in the concentrations of glucose to levels higher than 350 mg/dL have been shown to downregulate GLUT4 transporters and glucose uptake in skeletal muscle [279], suggesting an interplay between hyperglycemia and glucose transport to protect this tissue from glucotoxicity if circulating glucose exceeds physiological limits. 

Regarding the liver, hyperglycemia increases the rates of glucose uptake and rapidly suppresses glycogenolysis and hepatic glucose production, thus facilitating an increase in glycogen synthesis and glucose storage [280,281]. Studies in skeletal muscle by Mandarino et al. [282], using euglycemic–hyperinsulinemic clamps combined with biopsies, showed that, in the presence of basal concentrations of insulin (~10 μU/mL), hyperglycemia (~200 mg/dL) increased the rates of glucose uptake and phosphorylation. The increase in glucose 6-phosphate: (a) stimulated the activity of glycogen synthase by both an allosteric mechanism and by increasing substrate availability, and (b) increased the rates of glycolysis and glucose oxidation by stimulating the activity of pyruvate dehydrogenase. At higher concentrations of insulin (~60 μU/mL), hyperglycemia had effects additive to those of insulin to increase the rates of glycogen synthesis: glycogen synthase was activated directly by insulin and by a further increase in substrate availability and glucose 6-phosphate levels induced by high glucose levels [282]. In contrast, the rates of glucose oxidation were not increased more than by hyperinsulinemia alone [282] since, at these levels of insulin, the glucose oxidation pathway comes to saturation and glucose storage represents the major route of glucose disposal [148]. Thus, as recently suggested by Kowalski et al. [155], from a teleological perspective, the liver may permit a subtle increase in blood glucose levels after a meal (normally up to 140–160 mg/dL) to collaborate with insulin; this will minimize the amount of insulin required for optimal regulation of postprandial glycemia and reduce the secretory burden on β-cells.

### 4.9. The Role of Meal Sequence and Composition

#### 4.9.1. Meal Sequence within the Day

The sequence of meals plays an important role in postprandial glycemic responses: (a) in healthy humans, Bonuccelli et al. [283] showed that administration of two sequential equal glucose loads separated by 3 h was associated with attenuated glycemic responses after the second load, although insulin secretion rates and plasma insulin levels were lower than after the first load. Regarding the mechanisms, the suppression of endogenous glucose production achieved by the hyperinsulinemia and hyperglycemia of the first glucose load was maintained and strengthened by the subsequent load; plasma levels of GLP-1 were higher after glucose reloading [283]. (b) In support of these findings, Jakubowicz et al. [284] investigated the effects of absence or presence of breakfast (08:00) on postprandial hyperglycemia after identical meals given for lunch (13:00) and dinner (19:00) in subjects with type 2 diabetes. Interestingly, skipping breakfast worsened postprandial hyperglycemia not only after lunch but also after dinner. The omission of breakfast was associated with lower insulin secretion and GLP-1 responses, and higher plasma glucagon and NEFA levels after lunch and dinner compared with when breakfast was consumed. Although glucose turnovers were not measured in this study, these authors [284] hypothesized that the increased plasma NEFA and glucagon levels after lunch and dinner should have attenuated the expected suppression of endogenous glucose production and increase of muscle glucose uptake. (c) To separate the effects of isolated hyperinsulinemia or hyperglycemia during the consumption of breakfast on hepatic glucose handling during a subsequent meal later in the day, Moore et al. [285] performed 4-hour euglycemic–hyperinsulinemic or hyperglycemic–euinsulinemic clamps in conscious dogs using somatostatin and tracer infusions in the morning followed by combined hyperglycemic/hyperinsulinemic clamps later in the afternoon to mimic the changes in blood glucose and insulin seen after the consumption of a meal. The results showed that morning hyperinsulinemia but not hyperglycemia enhanced hepatic glucose uptake during the combined hyperglycemic/hyperinsulinemic clamp in the afternoon by augmenting glycogen synthesis and reducing endogenous glucose production. The authors [285] concluded that morning hyperinsulinemia is the factor to prime the liver to extract and store glucose more efficiently during subsequent meals, supporting previous findings in humans [283,284]. (d) Magnusson et al. [136] and Shulman et al. [139] gave either an oral glucose load or a solid mixed meal to healthy subjects twice: the first time the glucose load or mixed meal was consumed after an overnight fast and the second time later in the same day. Tracer infusions permitted the estimation of hepatic glucose uptake. The results showed that, after the first meal, about 50% of glucose stored in liver glycogen was by the direct pathway (first pass); this was increased to 70% after the second meal later in the day. Therefore, preceding meals may sensitize the metabolic and incretin system to the following ones, thereby improving glucose tolerance during the day mostly by increasing glycogen storage in the liver.

#### 4.9.2. Nutrient Sequence within the Meal

Postprandial glucose and insulin excursions can also be modulated by the premeal ingestion of noncarbohydrate macronutrients, such as protein and fat either alone or in combination: (a) Trico et al. [286] gave 75-g oral glucose loads enriched with [U-^13^C] glucose to trace glucose absorption from the gastrointestinal tract. Thirty minutes prior to the oral glucose loads the subjects received a small solid mixed protein/fat meal (23 g protein, 17 g fat, and 2 g carbohydrate, 1000 Kcal). The mixed preload increased insulin secretion rates and plasma insulin levels after the oral glucose ingestion (mostly during the first 30 min), and lowered glucose responses. The rate of glucose absorption was decreased, and plasma GIP/GLP-1 levels were increased after the oral glucose when the mixed preload was given. These authors [286] concluded that administration of a protein/lipid preload can slow down the rates of glucose absorption from the gastrointestinal tract and hence improve postprandial β-cell function during main meals. Interestingly, these effects were more marked in subjects with impaired glucose tolerance and type 2 diabetes, suggesting a useful therapeutic intervention to improve glycemic control. (b) Gentilcore et al. [287] investigated the effects of fat consumption (olive oil) 30 min prior to a carbohydrate meal (mashed potato) on gastric emptying, and glycemic, insulin, and incretin responses in subjects with type 2 diabetes. Fat preload markedly slowed the rates of gastric emptying and attenuated the postprandial increases in glucose, insulin, and GIP, whereas GLP-1 secretion was stimulated after the carbohydrate meal. As discussed by the authors, the stimulation of GLP-1 might have contributed to the reduction in glycemia by both slowing gastric emptying and stimulating insulin secretion [287]. (c) Ma et al. [288] examined the effects of protein (whey) consumption 30 min before a carbohydrate meal on gastric emptying and glucose/insulin/incretin responses in subjects with type 2 diabetes. Administration of the protein preload markedly reduced postprandial hyperglycemia and increased plasma GIP and insulin levels whereas increased GLP-1 secretion after the carbohydrate meal. The increase in insulin secretion after a similar carbohydrate meal was more than that observed in a previous study by the same group of authors when fat was given as a preload instead of protein [287]; this can be explained by the direct stimulation of β-cell secretion by the amino acids absorbed following protein digestion [265]. (d) Shukla et al. [289] examined the order of macronutrient consumption as parts of a mixed meal in subjects with type 2 diabetes. The subjects consumed an isocaloric meal (55 g protein, 68 g carbohydrate, 16 g fat, 628 Kcal) on two separate visits. In the first visit, the food order was carbohydrate followed 15 min later by protein and fat. In the second visit, the food order was reversed. Postprandial plasma glucose and insulin responses (incremental areas under the curve) were decreased by 73% and ~50%, respectively, when protein and fat were consumed first [289]. (e) The effect of food intake sequence on postprandial glucose, insulin, and incretin responses has also been investigated in healthy subjects by Sun et al. [290]. The subjects received isocaloric mixed meals with vegetables, meat, and carbohydrate (rice) consumed either together or in different order. The ingestion of vegetables followed by protein and then carbohydrate decreased plasma glucose and insulin levels by ~32% and ~25%, respectively, and increased plasma GLP-1 levels by ~29% compared to when carbohydrate was consumed first. The authors [290] discuss the importance of dietary fiber in attenuating postprandial glycemic responses when consumed first [291].

Taken together, the results of these studies suggest that nutrient type and sequence are key regulators of postprandial hyperglycemia by a variety of mechanisms involving gastric emptying, intestinal glucose absorption, release of incretins, and insulin secretion and action (summarized by Nesti et al. in reference [292]).

#### 4.9.3. Meal Composition

Low glycemic index/load diets are associated with lower postprandial glucose and insulin responses [151,152,293,294]. Low carbohydrate diets have also been shown to improve the metabolic syndrome and insulin resistance in obese individuals independently of weight loss [295]. Interestingly, in healthy subjects, ingestion of high-glucose mixed meals (45% carbohydrate as glucose powder, 20% protein, and 35% fat) impaired skeletal muscle microvascular function for up to 2 h postprandially, although blood glucose and insulin responses were not far from what is normally expected after meals (about 145 mg/dL and 60 μU/mL, respectively) [296]. As outlined earlier in this review, insulin-stimulated increases of blood flow in peripheral tissues are important for the physiological regulation of glycemia in the postprandial period [64,69,70].

In a series of experiments, Meng et al. [297] assessed the effects of different amounts of carbohydrate, protein, and fat added to a standard test food (50 g carbohydrate, white bread) to explore the hypothesis that different amounts of additional macronutrients would affect the postprandial glycemic responses and, hence, alter measured meal glycemic index/load values and blood insulin/lipid responses. The results showed that glycemic responses were reduced, and measured meal glycemic index/load were lower compared with calculated meal glycemic index/load values when carbohydrate-containing foods are consumed in combination with protein but not with fat [297]. However, in a subsequent report, Trico and Natali [298] commented on these results pointing out that the absence of an effect of fat in the study by Meng et al. [297] could be due to the slower rate of absorption of this macronutrient compared to carbohydrates [111], and therefore its effects might have been missed over the 120 min duration of the experiments.

### 4.10. The Importance of Insulin Sensitivity

A final point to be discussed is the importance of insulin sensitivity in the regulation of postprandial glucose metabolism. The amount of insulin released from the β-cells under any circumstances relies upon the metabolic requirements of the insulin-sensitive tissues, namely liver, skeletal muscle, and adipose tissue [2]. Thus, if an increase in insulin sensitivity occurs, for example in muscle, the management of an increased entry of glucose into the bloodstream after a meal could be achieved in the absence of a marked change in the blood concentrations of insulin; blood glucose levels would be controlled by changes in insulin sensitivity at the tissue level rather by an increase in the rates of insulin secretion and hence in the blood insulin concentrations. In earlier studies using intravenous glucose tolerance tests and the minimal model technique, Bergman et al. [299] hypothesized that the relationship between insulin sensitivity and β-cells is governed by a closed-loop feedback system that would require reciprocal changes in insulin sensitivity and secretion to maintain glucose metabolism unchanged. Kahn et al. [300] provided experimental support to this hypothesis showing that the β-cells and insulin sensitive tissues interact in a tightly regulated manner: when insulin sensitivity is high, insulin release is low and vice versa. Within this context, lifestyle interventions, such as optimal regulation of body weight and increased physical activity/exercise, are important to improve the sensitivity of tissues to insulin, and hence avoid marked hyperglycemia and hyperinsulinemia under fasting or postprandial conditions [301,302,303,304,305].

## 5. Conclusions

Glucose levels in blood must be constantly maintained within a tight physiological range (fasting 70–80 mg/dL and postprandial 140–160 mg/dL) to provide fuel for vital tissues (such as the CNS) and sustain anabolism. Insulin plays a primary role in glucose homeostasis via its effects on insulin-sensitive tissues: blood levels of glucose are regulated simultaneously by the rates of glucose production from the liver (and kidneys), and the rates of its removal from peripheral tissues (mainly skeletal muscle). Adipose tissue is a key partner in the liver–muscle interplay, providing NEFA as an alternative fuel for skeletal muscle and liver, when blood glucose levels are depleted. In the postabsorptive and postprandial period, insulin works in close collaboration with glucagon and the other the anti-insulin hormones to maintain a normal fuel balance between tissues and achieve glucose homeostasis. Because insulin is a potent antilipolytic hormone, decreased blood levels during the postabsorptive (fasting) period will increase adipose tissue lipolysis and blood NEFA; these will reduce glucose uptake in muscle and mediate the increase in endogenous glucose production to maintain blood glucose levels within the normal range. This is particularly important during sleep, in which anti-insulin hormones gradually induce a state of insulin resistance that peaks at dawn. The postprandial state puts a considerable burden on metabolism since the tissues must store and utilize the ingested glucose. This is achieved by an integrated interplay between food intake, meal sequence, gastric emptying/intestinal glucose absorption, gastrointestinal hormones, neural pathways, insulin/glucagon secretion and action, and glucose disposal. Increased concentrations of insulin in the postprandial period decrease adipose tissue lipolysis, resulting in a decrease in blood NEFA levels, which then: (a) mediate the decrease in endogenous glucose production, permitting the initiation of glucose storage, and the increase in glucose uptake by skeletal muscle and (b) help to incorporate the lipids of the meal into the adipose tissue triacylglycerol stores; this is facilitated by the insulin-mediated increase in adipose tissue blood flow. The contribution of the liver to the postprandial glucose homeostasis is critical; the liver is preferentially used to dispose over 50% of the ingested glucose, thus allowing a much smaller amount to escape to the peripheral circulation to be removed by muscle, and hence protect the circulation from the adverse effects of marked hyperglycemia and hyperinsulinemia. The regulation of postprandial glucose handling is extremely complex, and the numerous factors involved do not operate individually; rather, they function in concert, often promoting the simultaneous secretion of their antagonists to balance their effects. The interplay between all implicated mechanisms ensures a finely tuned adjustment of nutrient supply to the metabolic needs under all circumstances.

## Data Availability

Not applicable.
